# Beyond the smart city: a typology of platform urbanism

**DOI:** 10.1186/s42854-022-00033-9

**Published:** 2022-03-26

**Authors:** Federico Caprotti, I.-Chun Catherine Chang, Simon Joss

**Affiliations:** 1 Department of Geography, University of Exeter, Mail Room, The Old Library, Prince of Wales Road, Exeter, EX4 4SB UK; 2 Department of Geography, Macalester College, 1600 Grand Ave, Saint Paul, MN 55105-1801 USA; 3 Urban Studies, School of Social and Political Sciences, University of Glasgow, 25 - 29 Bute Gardens, Glasgow, G12 8RS UK

**Keywords:** Platform urbanism, Smart city, Digital city, Intermediation, Urban futures, Digital platforms

## Abstract

Platform urbanism has emerged in recent years as an area of research into the ways in which digital platforms are increasingly central to the governance, economy, experience, and understanding of the city. In the paper, we argue that platform urbanism is an evolution of the smart city, constituted by novel, digitally-enabled socio-technical assemblages that enable new forms of social, economic and political intermediation. We offer a typological framework for a better conceptualization of platform urbanism and its complex socio-economic relationships. We further outline several directions for future research on platform urbanism, specifically: a.) the need to critically investigate new power geometries of corporate, legal and regulatory alignments; b.) how platform urbanism may be expressed in, and affect, cities in the Global South; c.) how it may need to be critically engaged with in regard to its development in response to emergent events such as the Covid-19 pandemic; and d.) how it may shape visions of the current and future city.

## Scientific highlights

Platform urbanism refers to a novel set of digitally-enabled socio-technological assemblages rooted in the urban, which enables the emergence of new social and material relationships including intermediations and transactions.

The research adds a typological understanding to current work on platform urbanism.

Platform urbanism is an evolution beyond the smart city, based on digitally-enabled assemblages that enable novel forms of intermediation.

Platform urbanism is typologically understood in relation to the assemblage of actors involved in each platform type, and the platform’s connection to the urban.

## Policy and practice recommendations

Strategies to digitally transform the city need to take into account the implications of different types of platforms and the specific geometry of their constituent actors.

There is a need for digital urban policies to be critically aware of how platforms can reshape the nexus between corporations, Cities, and citizens

Future research on platform urbanism needs to explore how urban platforms may function and be shaped differently in Global South contexts.

## Introduction: digital platforms and the city

Recent years have seen a significant evolution in the content and trajectory of emergent themes in urban futures research, from the eco city and low-carbon city of the 1990s and early 2000s (Joss 2015), to digital cities and the smart city in the 2010s, and more in between (de Jong et al*.* 2015). What transpires, increasingly, is a vision of the urban future as technologically complex. Previous leading imperatives (e.g., environmentalism, resilience, equity, and justice) are now included within an envelope of technical solutions enabled by real-time data analytics and Artificial Intelligence (AI), which are deemed as systemic, fluid and efficient. While previous leading urban development trajectories are not being elided, they are now enmeshed within a broader network that has a distinctly digital and technical focus. At the same time, the services, products and relationships facilitated by the emergent digitally-enhanced city are mediated through socio-technical assemblages commonly referred to as ‘platforms’.

In this paper, we explore platform urbanism as an emergent phenomenon where technological platforms are rapidly taking center stage in shaping new visions, discourses, practices and materialities of the urban future (Barns 2020; Caprotti and Liu 2019; Rose et al 2020). Specifically, we chart the connections between the smart city and urban platforms, and relate them to the development of the contemporary global economic system towards what has been termed platform capitalism (Srnicek 2017). Although there are overlaps in scope and definition between the two (see Table 1), we argue that platform urbanism goes beyond the smart city as it signifies a key shift towards data-centered digital systems that are purposefully designed as templates to be applicable across multiple towns and cities. The smart city can be characterized as concerned with the city per se and with its various components, such as infrastructure, utilities and governance systems. Its premise is that these components can be digitally retrofitted, upgraded, and even ‘twinned’ (Winter and Tomko 2019). The main actors in the smart city field are municipal authorities, technology corporations and research organizations, working in partnership to achieve city-specific innovations and urban change (hence the common conjunction of the ‘smart’ moniker with a city name, such as ‘Smart Dubai’ or ‘Smart Nation Singapore’). In turn, the services and functions enabled by the smart city are city-specific, aimed at its citizens, visitors, businesses, civic organizations and public institutions. Platform urbanism, on the other hand, is not primarily defined and delineated by set urban geographies, but rather by novel, digitally-enabled assemblages (Dalton 2019; Kitchin and Lauriault 2018) of technology firms, providers of goods and services, users/consumers, and the resulting intermediations (Barns 2020) centered upon bespoke transactions. These range broadly, from private ride-hailing (e.g. Uber) to meal delivery services (e.g. Deliveroo) and from lodging (e.g. Airbnb) to active mobility services (e.g. Mobike). Insofar as these socio-technical assemblages rely on dense infrastructures and networks of actors, they are prevalent in the urban space: they draw on, and intervene in this space, although are not fully bounded by it. While a majority of urban platforms, resulting from technological entrepreneurship, are designed for commercial/monetized transactions, some recent examples entail public functions (e.g. Smart Health TeleRehab; Singapore) orchestrated by city authorities. Hence, the boundaries (conceptual, functional, spatial) between the smart city and platform urbanism also partially overlap.

**Table 1:** Key differences between the smart city and platform urbanism

Comparative dimensions	Smart city	Platform urbanism
Area of focus	**The city itself.** While the concept may be generic (aimed at policy transfer/mobility), it is primarily understood in terms of ‘improving’ an existing city through retrofitting and adding a digital (infrastructure) layer. While the notion of ‘city’ itself is being challenged, the smart city typically relates to materially-recognizable cities. This can be seen in the proliferation of smart city league tables (Albino et al 2015). The key focus of smart urbanism is: *a city*	**The platform, as a socio-technical assemblage**. Platform infrastructures rely on dense assemblages (and hence are particularly suited for urban contexts): large number of providers (e.g. taxi drivers, apartment owners, restaurants), and users (residents, workers, visitors, consumers), and technology firms. Typically, a platform is constructed to be scalable to any, or multiple, urban environments. The key focus of platform urbanism is: *any city*
Rationale	**City hall perspective**: to ‘upgrade’ the city, to bring greater benefits to residents and to render the city more competitive internationally. Also, against the background of austerity, to make utilities and services more cost-effective **Corporate perspective**: to take advantage of increased urbanisation to sell digital systems and technologies to municipalities. The city/urban is seen as a significant growth market (Caprotti and Cowley 2019)	**Platform provider perspective:** to offer an attractive, digitally enabled interface between service producers and consumers, which can be layered upon, embedded in, and applied across multiple urban contexts. To offer opportunity for investment and stakeholder returns, based on a scalable business model **Platform user perspective**: to obtain/consume services through an integrated portal and in prompt, convenient fashion; to engage in complementary social interactions
Spatiality	**Bounded**: relating to a geographically recognizable, demarcated (if increasingly blurred) area. Even in abstract discussion, the concept mostly refers to the city as a geographical area and governance entity. Furthermore, while the smart city exhibits policy mobility, mobility is discussed in relation to transferable knowledge/practices/lessons from specific cities (Wiig 2015)	**Porous and extendable:** The platform is territorially defined by the interaction between service providers and users, plus the availability of the underlying technological system. As such it is characterized by geographical and temporal flexibility and fluidity, in turn influenced by urban density. From a user perspective, the platform can be used across different cities (e.g. Uber, Airbnb), whereas a smart city app (e.g. London’s Oyster card) can only be used within the designated city/service
Temporality	**Long-term.** Smart city investment is fundamental in nature: upgrading infrastructure, improving administrative and governance systems, etc. As smart city policies, strategies and projects ultimately originate from city hall (see below), the smart city’s temporality is at least partly defined by electoral cycles and government periods. While the smart city produces real-time data for instantaneous planning (e.g. traffic management), it is also used for *longitudinal analysis* to inform long-term planning	**Contemporaneous, short-term, instantaneous.** The focus is on day-to-day services and interactions, which change and evolve based on market forces. An e-bike scheme may quickly ‘flood’ the city (as part of marketing strategy), but also disappear again depending on market demand and social resonance. However, platforms draw and rely upon long-term and permanent infrastructure, built environment and technology
Governance	**City authorities** are the main driver of smart city activities, with a strong relationship between city hall and private technology corporations. The smart city may spawn diverse private initiatives and partnerships, but it typically functions under the municipal umbrella. Smart city innovation ultimately serves public governance: facilitating municipal administration, public service delivery, and the relationship between officialdom and citizens	**Platform operators self-defining as technology firms**, operating across cities with the involvement of local providers and users. Governance is based on legal contracts/relationships: partnership agreements with e.g. restaurants (Deliveroo) and property owners (Airbnb) and ‘workers’; as well as users/consumers (who agree to ‘terms and conditions’ of use). City authorities often become involved as regulators (e.g. London Mayor threatening to ban Uber over passenger safety concerns). It is worth noting that several (European) cities have banned specific platforms (e.g. Uber). Also, several court cases at national and European levels have arbitrated on whether platform providers are technology firms only (and therefore exempt from usual municipal regulations) or instead should be recognized/accountable as city service firms (i.e. a transport company in the case of Uber)
Technology	**Smart city technologies as in situ digital infrastructure:** interacting with, and between, other ‘fixed’ urban infrastructure and municipal administration, to provide improved *municipal* services (public transport; congestion charging; smart metering for water utilities; environmental data capture and reporting; online citizen dashboard, e-reporting and e-voting etc.)	**Digital technology and data analytics as platform backbones**. Real-time data, collected from ‘partners’ (Uber drivers, Airbnb hosts etc.) and ‘users’ (passengers, visitors etc.) using a platform-specific app, is analysed to connect partners and users in the most optimal configurations. The platform provider’s technology infrastructure may be located a long distance away from the platform’s day-to-day urban application

Consequently, we define here platform urbanism as *‘urban development and urban life facilitated by a growing number of digitally-enabled, socio-technical assemblages that engender new kinds of social, economic and political intermediations’*. It is interrelated with the emergence of platform capitalism, a new business model premised on the extraction and control of vast amounts of data and favoring large monopolistic firms (Srnicek 2017: 5). How platform capitalism circulates and ‘lands’ in cities and urban areas worldwide, and potentially changes the nature of the urban experience, is therefore essential to our understanding of platform urbanism. To what extent do the platforms display a specific focus on the city? And in what ways do platforms redefine or even challenge the ways in which the urban is understood? This paper seeks to provide answers to these key questions, first, by tracing the roots of platform urbanism in both the smart city and platform capitalism literature and considering their foundational contributions so far. We further conceptualize platform urbanism from the twin perspectives of socio-technical assemblage and intermediation, before discussing a number of possible configurations of how platform urbanism dynamically draws on, and intervenes in, the urban. This leads us to conclude with some suggestions for future conceptual and empirical research, focused on the need to critically engage with new power geometries emerging as a result of platform urbanism, the expression of platform urbanism within Global South urbanism, its relation to the recent Covid-19 pandemic, and its role in shaping visions for current and future cities.

## From the smart city and platform capitalism to platform urbanism

As both a concept and practice, platform urbanism is genealogically rooted in the rapid and multifaceted emergence of smart urbanism over the past decade (Hollands 2008). The smart city is generally understood as an urban environment characterized by the use of Big Data, digital flows, and networked technologies (Kitchin 2014; Townsend 2013), as well as by experimental approaches to the use of these technologies (Luque-Ayala and Marvin 2015; Tironi and Valderrama 2018), in terms of both governance (Cowley et al 2018; Cowley and Caprotti 2018) and urban and city-regional economic development (Caragliu et al. 2011). Many scholars have highlighted the difficulties inherent in trying to define the smart city (Crivello 2015; Hollands 2015), although recent work has moved past this conceptual quagmire by highlighting the emergence of a diverse landscape of smart and ‘ordinary’ cities (Karvonen et al 2018; Shelton et al 2015). In particular, recent work has pointed to how a focus on the materialities and local practices, spaces, and infrastructures of specific cities helps to move past definitional debates and towards grounded engagement with existing smart urban environments (Gabrys 2014). All of these aspects of smart urbanism (from the centrality of data, to the role of networks, infrastructures, and digital materialities in real- or near-real-time) contribute to, but do not fully capture, the nature of platform urbanism. We understand this phenomenon as a contemporary, data-enabled and networked undergirding and intermediation of urban life: ‘our urban space is…underpinned by a blurred and complex platform-based ecosystem encompassing public and private organisations and people/citizens’ (van der Graaf and Ballon 2018, np). Thus, platform urbanism’s central focus is on leveraging existing and/or new data-centred intermediations to *assemble* (or reassemble) and *configure* (or reconfigure) things, relationships, actors, technologies and urban systems into different, sometimes new, geometries.

While platform urbanism draws on the smart city in its conjunction of the digital with the urban, research on platforms in contemporary society is rooted in Srnicek’s (2017) work on platform capitalism. Srnicek argues that contemporary capitalism is undergoing a profound shift as a result of crises in manufacturing profitability on the one hand, and the rise of the digital, knowledge-based economy on the other. A key aspect of this change is that data has become ever more central to the economy and to its organization, and that the *platform* has emerged as the key mechanism and device. The ‘platform’, then, describes the ‘set of online digital arrangements whose algorithms serve to organize and structure economic and social activity’ (Murillo et al 2017, 67). Indeed, while the factory and the superstore were emblematic of twentieth century industrial capitalism, platform capitalism is increasingly populated by large corporations (from Amazon to Alibaba, to Facebook and Google and many in between) whose role it is to extract and exploit data from consumers (Wagner, 2021). Platforms’ key weight is therefore in enabling intermediation (between consumers, producers, and other actors) (Langley and Leyshon 2017a). At the same time, its growth is dependent on capitalization potential, that is, the speculative practice of leveraging debt from investors based on potential future returns (Langley and Leyshon 2017a) accruing to platforms that become near-hegemonic.

Platform capitalism displays a key focus on the city (Gillespie 2010). The city serves as both an inspiration and testing ground for the development of platform applications. An example of this is the development of the Deliveroo meal-delivery service in London, before its roll-out across UK and to international cities. Similarly, Uber was initially tested in San Francisco in 2010, before its eventual spread nationally. Although some platforms commonly discussed in the literature (e.g., Airbnb, Uber, Amazon, Deliveroo, Taskrabbit) are not foundationally urban in scope, they indeed are the key financial intermediaries for other urban platforms to operate monetary or other transactions or exchanges. These platforms are rooted to a great extent in the city as the spatial underpinning of the performance of platform-based economies; the business models, use cases, and pragmatic rationales for the platform are embedded in the socio-economic, spatial and cultural characteristics of cities. For example, while mobilities platforms are not explicitly defined as urban by the corporations that develop them, at the same time their business models rest on the ability of the platform to leverage the customer and provider base that is characteristic of an urban environment vis-à-vis a more rural or dispersed setting. Thus, platforms are urban not necessarily because of their being designed with a specific city focus. Rather, they are designed to behave as though their operational environment is urban. This underscores the need for a specifically urban analysis of city-specific expression(s) of platform capitalism at different scales. These range from the individual urban citizen using smartphone-based apps to access and inform governance-related information, to corporate platform services such as ride-hailing services, to city planning departments interfacing with AI and data analytics services (such as Alibaba’s CityBrain systems in use in several Chinese cities) (Caprotti and Liu 2019, 2020); to platforms such as food delivery app Deliveroo, or property rental platform Airbnb, that interact with the labour market in the former case, and with the valuation and monetization of fixed assets on the other (Boeing et al., 2021), and thus impact on the urban economy.

Platform capitalism represents a move towards an “ideological imaginary” associated with the reconfiguration of production, consumption, distribution and monetization of goods and services (van der Graaf 2018: 153). This encapsulates platforms’ effects in reshaping and reconfiguring various facets of urban economic, cultural, and technological experience. It also allows platform urbanism to be the centre of platform economies, while extending the role of the platform beyond the confines of the smart city, with its organizing logics around ‘sharing’ and ‘smart’ (Barns 2019). Exactly because of its potential in reconfiguring diverse techno-social geometries, platforms urbanism has emerged in response to several key challenges: a) the need to urbanise data-based, algorithmic business models (Pollio 2021); b) the linked imperative to move beyond the smart city’s focus on the urban governance-technology corporation dyad and towards the nexus between capital, technology, cities and consumers through the platform (Sadowski 2020); c) the call to explore alternatives to the traditionally hierarchical and on-size-fits-all approaches in smart city governance (Leszczynski 2020). We therefore argue that platform urbanism is rooted in, but goes beyond, the smart city as it is currently understood (Table 1).

We pinpoint three aspects of platform urbanism that define its nature as separate from what is normally understood as a ‘smart city’. Firstly, platform urbanism is based on integrating flows of diverse data into specific platforms that offer (commercial or governance) services. At this level, it can be seen as a set of technological ‘solutions’ to specific ‘problems’ seemingly disconnected from one another. An example of the integration of data is WeChat, which combines messaging, social media, mobile payment and other functions (as well as the potential for the platform to integrate with other platforms). This data-integrative focus is, we argue, constitutive of a performance of cityness by urban platforms that is dynamic, extensive and non-normative (Brenner and Schmid 2013).

Secondly, platform urbanism is by definition applicable at both a city-specific and an inter-city scale. The technologies that make platforms useful systems can be applied both within specific cities, and made sensitive to local contexts, as well as being used in several different urban centres, often in different political jurisdictions. Thus, urban platforms are typically developed as generic socio-technical assemblage templates, with the purpose of applying them across multiple sites. This characteristic is deeply tied to the nature of the platform itself: as Nieborg and Helmond (2018) argue in their analysis of Facebook-as-platform, a platform can be segmented into separate ‘platform instances’ visible to specific user groups. In the case of urban platforms, this can be seen in the tailoring of specific platforms to local contexts. In bringing together data on different aspects of urban life applicable across cities and their hinterlands, urban platforms are integral to processes of planetary urbanization, when the latter is understood as a perception of broad urban change processes (Merrifield 2018). Platform urbanism enables not only the operation of specific platforms in the city, but also the extension of the city to non-urban settings. An example of this is the rise of so-called ‘Taobao villages’. These are formerly relatively poor rural hamlets that have developed thriving economies through their use of Taobao.com, a retail platform (Qi et al. 2019). The platform enables villagers to sell products in highly specialized formats and often for niche markets. At the same time, the villages function as an interface between the market (in the city) and producers (both of products and of the raw materials from which these are made). As Lin (2019) argues, the example of Taobao villages shows that platform urbanism operates through a layered construct of Information and Communications Technology (ICT) networks, social networks and relations, and urban form and land use. These layers do not overlap neatly in space. Some layers (notably, social and telecoms networks) have considerable spatial extent. The Taobao platform enables the bridging of urban and rural, effectively extending the digital shadow of the city into rural spaces and effecting economic change, and thus constituting a novel variant of the urbanization process.

Thirdly, we argue that one of platform urbanism’s key characteristics is its treatment of data as a resource for urban management and economies. Platforms rely on pervasive and ubiquitous data, as well as sophisticated analytics and advanced algorithms including AI. In turn, the centrality of data to the functioning and essence of urban platforms is related to the function of platforms within an era defined by platform capitalism. In this sense, integrating multiple forms of data is key because the provision of services and goods through platforms is part and parcel of the capture and intermediation of data by platform operators. In turn, data capture and usage are linked to what has been termed ‘platform accumulation’, understood as a ‘deepening of forms of privatization, marketization, and corporate consolidation associated with neoliberal capitalism more broadly’ (Meier and Manzerolle 2018: 2). Ultimately, while ‘the platform is a powerful metaphor for the way contemporary society organizes and understands itself’ (van der Graaf 2018: 153), it is also an emerging reality in practice, as manifested in urban life in manifold ways. Platforms are therefore crucial for understanding future-focused urbanism in the contemporary city.

## A typology of platform urbanism

In order to move towards an understanding of the ways in which platform urbanism can be conceptualized, it is useful to build on, and expand, current understandings of the broader context of platform capitalism. Srnicek’s (2017) typology of platform capitalism is based on several analytical categories (advertising, cloud, industrial, product and lean platforms)^1^ to explore the reconfiguration of production, consumption, distribution and monetization of goods and services under platform capitalism. Several studies also have sought to apply the category notion of platform in order to understand the imbrication of data and established and emergent economic sectors, such as crowdfunding (Langley and Leyshon 2017a, 2017b), the education marketplace (Williamson 2017), skills marketplaces such as Linkedin (Komljenovic 2018), online content platforms (Gillespie 2010) and darknet-based illicit marketplaces (Dittus et al. 2018). Facing the growing complexity of the platform landscape, scholars have recently called for efforts to ‘consider the underlying characteristics of platforms and business models rather than trying to deal with digital platforms as a single category’ characteristics of platforms (Nooren et al. 2018, 267). Inspired by such call, we develop a typology of platform urbanism to assist our analytical understanding of new trends in urban development.

The typology outlined below attempts to encompass the multiple configurations of the emergence and establishment of urban platforms. Proposing a broad typology helps to map the varied landscape of platform urbanism and reveal different operational logics (Barns 2018, 6). We also raise three qualifications that need to be considered prior to offering our typology. Firstly, we do not claim that the typology is to be considered fixed, but rather as an overview of a rapidly-emerging and changing field of practice, innovation, consumption and citizen engagement within the urban. This is a point recognized in the literature: Nieborg and Helmond (2018: 5) point out, for example, that while Facebook could be defined as both a business and a technical platform, ‘both dimensions are mutually constituted’ and can be considered a ‘double articulation’ of Facebook’s underlying platform logic.

Secondly, following Srnicek’s (2017) argument that the typology of platform capitalism should be seen as interactive rather than as consisting of strict binaries, the urban typology discussed here should not be seen as an attempt to ossify our understanding of platform urbanism into conceptual silos. Instead, the typology we develop should be considered a potential framework for analysis. This framework is intended to encapsulate the dynamic nature of the assemblages that constitute platform urbanism. Using the framework for understanding intermediation in transitional pathways developed by Kanda et al. (2020), our focus on the intermediary nature of categories within our typology contributes to understanding how entities interact within assemblages. Additionally, it is key to note that intermediation may occur between different typological categories. There is also the potential for dynamic intermediation to occur between categories in such a way that broader, systemic effects are generated. Finally, it is key to note that an actor within a specific intermediation relation, nested within a single typological category, can also be active in other intermediary relationships that stretch across, and connect to, other actors and networks in different categories and scales.

Third, the typology developed for this paper does not preclude the need for further work on the specific dimensions and typologies of distinct types of platforms. In other words, our work focuses on conceptualising platform urbanism, and we look forward to future research that excavates each part of the typology more deeply (or indeed, offers new typological insights). For example, in her work on data platforms, Barns (2018) usefully offers a typology of data platforms as data snapshots, data stores, and scorecards. Our typological analysis adds to Barns’ (2018) work in two ways: firstly we focus on an agential landscape of platform urbanism, and thus produce a more granular typological understanding of the multiple pathways in which platform urbanism as a phenomenon is currently expressed. Secondly, rather than focusing purely on data, the typology below is centred on the platform proper. It reconciles actor networks, technologies, and their material and geographical effects into the key construct of the urban platform. Building on this, the typology we offer is presented below. It is focused on four distinct categories, of which the first two are primarily private sector-focused, while the third and fourth are centred on the public and non-profit sectors.

### Type I: Online-to-offline producer–consumer intermediation

This category involves platforms functioning as intermediaries between producers of goods and consumers/customers, via distributors. Customers select goods digitally via the platform’s commercial interface, goods are prepared by private sector firms, and delivered via usually independent distributors. There is minimal involvement of public authorities, apart from the need for producers and distributors to comply with specific regulations and policies. Examples of this category are food delivery platforms such as Deliveroo or Meituan. Customers select meals on an app from a provider (usually a restaurant) that then prepares and deliver the food to the customers for consumption. Food safety and other regulations apply to the businesses that benefit from this type of platform intermediation. In this category, the city is key because of the spatial economy of urban aggregation and density that makes online-to-offline producer–consumer intermediation function from a commercial and user perspective. The city provides the density (of consumers, demand, and digital and market data) that gives these platforms their urban logic.

### Type II: Service provider-customer intermediation

Type II urban platforms are focused on intermediating between the providers of specific services, and customers who use digital, commercial platform interfaces to search for, and pay, for those services. In this category, the involvement of municipal and state authorities is usually in the role of regulators. Examples of this type of platform are ride-hailing forms such as Uber, where service providers (taxi drivers) and customers are placed in contact via a platform app; at the same time, providers have to navigate municipal and state regulations in order to be able to operate. Some of the firms that rely on Type II platform intermediation may not actually derive an income stream directly from the provision of a specific service, but through revenues linked to advertising. This is the case with the Google-owned Waze Global Positioning System (GPS) navigation software app. It provides navigation and traffic services for users by leveraging both Google’s mapping data, and users’ own broadcast positions, speeds and updates. Thus, users themselves provide part of the service intermediated by the platform, in the form of real-time traffic data (van der Graaf 2018). Meanwhile, Waze generates revenues not through making users pay for the service, but through location-based advertising: it is here that the urban specificity (the specific city context) of the platform can be seen as key.

### Type III: Public service intermediation

In this category, platforms fulfil the function of intermediating between public agencies (who provide a service), and the customer. In Type III, municipal authorities are central actors in both providing services and determining platform functionalities and the parameters within which service offerings operate. We have chosen to describe the beneficiaries or recipients of Type III platform services as customers rather than citizens because in some cases these platforms can be used, and may indeed be wholly aimed at, individuals not benefiting from citizenship status (e.g. in the use of platforms through which non-citizen residents are governed). Customers may also not be individuals, but public sector departments or agencies, or private sector firms. An example of Type III intermediation is the emergence of digital twins such as Virtual Singapore (Liceras 2019): data-based and temporally dynamic and self-updated representations, counterparts or cybernetic imaginaries (Barns 2020) of urban spaces (even whole cities) (Winter and Tomko 2019). Virtual Singapore is a digital counterpart that is aimed at being used by public and private sector users and customers: information about the city is intermediated through French digital technology firm Dassault Système’s 3DEXPERIENCE platform.

### Type IV: Not-for-profit service intermediation

This type involves the use of platforms as intermediaries between usually non-governmental, not-for-profit providers of services, and members of the public. Non-profit actors may include both Non-Governmental Organisations (NGOs), and civic service providers. An example of the latter is the provision of real-time public transport and routing information through platforms such as those used by many cities’ public transport authorities; or the provision of free environmental and weather data by national meteorological organisations or citizen sensing projects (Gabrys 2017). For example, Sustrans, a UK sustainable transport NGO, partnered with the UK’s Ordnance Survey (OS) mapping and survey authority to produce a new mapping layer on OS maps. The new layer makes the UK’s National Cycle Network visible to users of OS digital maps, through the OS app or on the web.

## Towards a conceptual understanding of platform urbanism

The typology and examples above illustrate the ways in which platform urbanism is expressed and operationalized in different urban spheres. Furthermore, the examples in each typology show how platform urbanism is a development of smart urbanism (as argued above) while at the same time stretching the remit of the smart city to include a broader range of economic, industrial, cultural and other phenomena that can be brought to bear on the city via the platform. Thus, we can offer an initial conceptual understanding of platform urbanism along the following lines:

### Hybrid agency

Firstly, platform urbanism involves a spectrum of agency: the agency of public, corporate and hybrid, public-corporate actor networks that can develop, use, and extend the spread of specific platforms. These networks are typically ‘at a distance’, not confined to specific cities, but transcending them. It is also clear that most of the typological categories explored above exist along a corporate-public axis: thus, platform urbanism encapsulates aspects of urban life that are purely corporate (such as retail platforms), or purely public (such as certain types of e-governance platforms), and examples that are a mixture of the two or that are expected to move along that axis (Sesame Credit, a Chinese corporate-led Social Credit System, exemplifies this). Most research on the smart city has highlighted the corporate (Hollands 2015; McNeill 2015) or state-led (Cowley et al. 2018) nature of many smart urban developments and projects. Our focus on the agency of platform urbanism extends this by excavating the complex ways in which platforms are assembled, involving a wide range of processes that are economic, industrial, logistical, knowledge-based, and legally and institutionally-framed across various geographical scales.^2^ Indeed, urban platforms often scale out and up both nationally and internationally. At the same time, the hybrid networks that characterize platform urbanism are also dynamic, involving a constellation of actors that changes across space and time. The temporal nature of these dynamic networks of platform urbanism is also key: much of the emergent literature on urban platforms has tended to understand them through a ‘snapshot’ or ‘freeze-frame’ approach. We argue that an approach to platform urbanism that is sensitive to its hybrid nature also needs to take into account the temporal scale along which platforms evolve through changes in their actor-network. For example, a platform innovator corporation may ‘flood’ a specific urban market in order to establish themselves and generate demand as well as gain market share, but may just as quickly withdraw from a city, with consequences for the urban area.

### The spatiality of platforms

Platform urbanism is deeply spatial, because while platforms may exist across boundaries and in very different urban, national, political and economic-regulatory contexts, nonetheless the platforms are grounded in specific urban realities at the city, neighbourhood, and street level. While Deliveroo may be a food-delivery platform active in Bristol and Hong Kong, for example, the specific commodity-focused economic exchanges enabled by the platform are bound to a spatially-specific nodes (e.g., the individual who orders food for delivery to a specific address within those cities). However, the actual financial exchange happens digitally, through a credit card-based interface within the platform: this element of the exchange enabled by the platform happens with relation to the fixed places of credit card corporations, servers, and Deliveroo’s own financial geographies. Thus, the specific geographical place where exchange occurs is both key (because platforms enable a link between these places and the broader digital economy) and part and parcel of broader, relational economic and cultural processes. It is in this sense that platform urbanism enables a view of the city that is both concentrated and dispersed in terms of its spatiality (Jordan 2015). This belies the notion that digital and smart technologies enable abstraction from space. Rather, it is evident that platforms function *across* space, but are rooted *in* place.

### Materiality and infrastructure

Following on from the discussion above, platform urbanism is not only highly spatially configured, but also deeply material. It is key for research agendas on platform urbanism to acknowledge this upfront. The focus of much of the literature on smart and digital urbanism has rightly focused on the increasing importance of code, data flows (Kitchin 2014), Big Data, and the trend towards what has been called ‘planetary-scale computing’ (Bratton 2016, 305). Nonetheless (and as seen in the Deliveroo example above), the economic activities enabled by platform urbanism are rooted in materiality. Deliveroo cannot exist without food, bikes, the human body. Uber cannot exist without cars, steel, asphalt, and the need for bodies to move from one place to another. Amazon cannot exist without the need for specific goods to be delivered to specific places. Even a movie or Kindle purchase on Amazon is material, in that the digital package is delivered to a physical device, in a specific location. In the case of bitcoin, likewise, the digital bitcoin economy is predicated on servers and plentiful supplies of electricity.

While code, servers and digital know-how are crucial to the business cases and operations of platforms, there are often banal and very widespread networks of infrastructures that make these platforms possible. Platform infrastructures often exist in the background, and are not visible on the high street or on digital interfaces. They do the work of connecting physical goods and services to their digital representation online, and it is here that platform urbanism can be said to become an urbanism of *spectacle*, understood in Debord’s (1994) terms as a social relation mediated by images. Amazon, for example, exists as an online platform but its physical backbone is an infrastructural assemblage of out-of-town distribution and fulfilment centres, storage, and logistics and delivery networks, as evidenced by Amazon’s 40-strong (at time of writing) and growing fleet of cargo planes (Cameron 2019).

## Discussion and conclusion: directions for a research agenda on platform urbanism

We premised the paper around two key questions: a) to what extent do the platforms under consideration display a specific focus on the city? and b) how do platforms redefine or even challenge the way in which the urban is understood? Our typological analysis shows that while platforms display generic fields of action that may, at first sight, not appear to be specifically tied to the urban, closer inspection shows that the city (usually, a specific city, at least initially) functions as both the inspiration, experimental site, and marketplace for these platforms. Thus, platform urbanism is clearly rooted in the urban at a variety of scales, and specific platforms are predicated both on the city as a generic field of action and upscaling, and on the city as a *place* in which platforms find their root. Secondly, it is clear that platform urbanism both extends, and in some cases challenges, an (the?) understanding of the urban as limited to specific cities or urban boundaries. The case of digital twins or shadows highlights platforms’ creation of digital dimensions of the urban, while the example of Taobao villages exemplifies the platform-enabled extension of the urban into contexts traditionally understood as completely rural. Platform urbanism therefore comprises novel dimensions that require further research and analysis.

This paper has explored, typologically and conceptually, a (the?) quickly emerging, dynamic, and agentially- and materially-complex landscape of urban platforms. We have also identified three conceptual dimensions of platform urbanism: hybrid agency, spatiality and materiality. Our typological and conceptual analysis has shown how platform urbanism, as a complex and at times highly unstable socio-technical assemblage, is characterized by hybridized form of agency that extends across and through the assemblage. This involves novel and sometimes transitory configurations of negotiated agency including private and public sector and other actors or actor networks. In turn, this agential dimension is expressed spatially, evolves over time, and may engage in geospatial dimensions not normally thought of as constituting the urban. In this sense, platform urbanism challenges some notions of what the ‘urban’ is, and what its sphere of operation and effects extends to. Additionally, the complex and extensive spatiality and assemblage nature of platform urbanism has implications in terms of materiality. From data centres in caves in central China, to fibre-optic cables on canal beds in Bristol, to the resource extraction and exploitation involved in sourcing key materials for smartphones, to knowledge economies around globally-mobile platform ‘talent’, platform urbanism exhibits specific material dimensions often belied by the focus on digital data and ephemeral digital geographies. Thus, we argue that platform urbanism effectively reworks the urban, reassembling it into novel configurations rooted in, but different from, earlier iterations of smart urbanism.

The implications of platform urbanism are multifaceted and necessitate further study. Through observing the current development of platform urbanism and considering the conceptual dimensions of agency, spatiality and materiality, we think it is important for future studies to engage with: a.) new power geometries of corporate, legal and regulatory alignments; b.) how platform urbanism may be expressed in, and affect, cities in the Global South; c.) how it may need to be critically engaged with in regard to its development in response to emergent events such as the Covid-19 pandemic; and d.) how it may shape visions of the current and future city. Here we offer our reflections and identify potential research agendas.

### Corporates, the platform and the city

Our first reflection focuses on the role of the private sector in shaping, steering and performing platform urbanism. Although there has been a years-long flurry of media attention on various urban and other platforms (from Uber to Alipay, from Airbnb to Deliveroo), questions need to be asked about the effect that platform urbanism is having, and will have, on cities in the near future. At the time of writing, there are emerging relational networks composed of urban authorities, the national state, and the private sector in designing and promoting platform urbanism. As McNeill (2017, 232–33, italics added) notes, ‘new tech duopolies will emerge to control the lion’s share of both the demand and supply sides of any *public* and private sectors that appear monetizable.’ The potential reshaping of private–public power geometries in the wake of platform urbanism is a key and emergent issue: new urban platforms can serve as interfaces not just between tech firms, governments and citizens in their instrumental use of such platforms. Rather, they can serve more broadly as interfaces between ways in which the relationship between private and public sectors is articulated and managed. Several recent studies point to the increasing tendency towards micro-control coupled with global technological hegemony displayed by several corporate platform corporations. These firms encapsulate within themselves a drive towards innovation coupled with a trend towards infrastructural control: the interplay between these two determinants has been called a ‘platform logic’ (Schwarz 2017, 378) in global political economy. It is crucial to critically engage with this and associated logics, as they form a challenge to existing balances of power between corporates and the state. Additionally, the rise of highly complex and capital-intensive platform corporations raises the spectre of a small number of technology corporations dictating the rules of engagement with and to cities and the state. Furthermore, even though it is crucial to focus on the ways in which power may be shifting to the corporate end of the spectrum, firms themselves are seeing a reshaping as a result of the rise of platform capitalism (Aloisi 2016, 2018). While our focus here is more specifically on the role of corporates in a corporate-public spectrum of agency, there are also key questions to be asked and researched with regards to how platform urbanism is reshaping the economic playing field.

Furthermore, another point of focus is the link between specific cities and the global technology and corporate networks active in promoting platforms that have urban impacts. For a city, these issues are deeply connected not simply to power geometries between local authorities and specific urban areas, but to the question of how to negotiate often different regulatory and policy environments. This is because single platforms operate across a range of these. Indeed, platform urbanism can be seen, to some extent, to have ‘created problems, largely as a result of the way social processes have been extracted from traditional (often nationally constrained) regulatory frameworks’ (Nash et al. 2017, 368). Cities can be seen to be ‘grappling with platform-focused questions such as how to limit the oversupply of guest accommodation in their cities, or [o]ther policy concerns, such as the need for just labor practices in the gig economy’ (Ibid, 368–9). At the same time, it is clear that some platform providers, at least, are showing signs of re-engaging with regulatory processes as a way of both gaining market access and political approval, and of exploiting potentially profitable niches. An example of this is Indian ride-hailing firm Ola, which started expansion into the UK in 2018. Its business model is based on aligning itself with regulatory requirements by applying for licensing from city authorities, so that licensed taxis are the vehicles that can be hailed using its service, rather than the Uber model in which in some cities, non-licensed vehicles can be hailed using the Uber app. This exemplifies Ola’s attempt to address current regulatory concerns around platform apps circumventing current regulations. On the other hand, regulatory alignment belies the exploitation of potential market niches: in the case of Ola, the app is being rolled out in some urban areas where Uber operates (Cardiff, Newport) as well as others that are seen as too small or dispersed to be attractive to Uber (Exeter, North Somerset, South Gloucestershire), but where profits can still be extracted.

When thinking about the role of corporates in platform urbanism, it is also key to be aware of broader, but no less important, questions around the impact of these new technologies on the city and its citizens. Urban platforms as they are emerging at the time of writing encapsulate a tension between public and individual goods, where by ‘public’ we denote a difficult to define sense of broader urban goals and interests over and above the ends of specific platform offerings. The corporate dimension is key in the context of this tension, because of the key role corporates and their design, beta testing and operationalization roles have in the interfacing of platforms and the city. This means that, as pointed to above, the power geometry between the push for individual goods through corporate-driven platform projects, and the need to govern cities in a context of rapid digital change needs critical attention. In the case of mobility platforms such as Waze, for example, van der Graaf and Ballon (2018) point out the complexities and urban side effects of increasing reliance on networked, real-time app-based mobility services, and the need to engage with the mechanisms of data generation, exploitation and governance more carefully. This is a key question in an era of Big Data-informed urban governance and marketisation (Riemens et al., 2021). Critical voices also point out that platform capitalism can be narrated from a variety of perspectives, from the efficiency-focused neoliberal celebration of platforms’ simplicity and efficiency, to critical voices’ focus on the abusive and exploitative practices that are often involved in the functioning of the platform economy (Pasquale 2016). This involves critiques of the ‘gig’ economy and its reliance on often low-paid and insecurely employed workforce (including its impacts on areas such as bargaining power and economic inclusion) (Graham et al 2017; Webster and Zhang, 2021), as well as analyses of how the ‘almost total failure of legal systems to hold capital to account’ (Snider 2018, 564) denotes a potential critical rupture in the state’s attempts to regulate capital. This point can be illustrated by considering the legal, corporate and agential complexity around the Deliveroo food delivery platform. The platform is owned by London-based Roofoods Ltd, and operates in more than 200 cities in 13 countries at the time of writing. A subsidiary corporation, Deliveroo Editions, focuses on establishing and running urban ‘ghost kitchens’ that prepare only food for delivery. These kitchens function as hubs for several different restaurants, and for Deliveroo riders who can pick up meals from a single hub. They utilize parts of the city (such as car parks or adapted buildings), and some (housed in temporary metal containers, many of them windowless) are potentially temporary and transient (Butler 2017). Customers purchasing meals made in these ‘ghost kitchens’ participate in the fetishism of the meal as a commodity, where relations of production, and the sites of production themselves, are masked by the fetishistic nature of the food delivery app itself, and of the restaurants’ own brand image(s). Additionally, part of the Deliveroo assemblage are restaurants themselves, who are effectively partners paying a commission to the corporations. Delivery riders, in turn, are self-employed (and often participants in fragmented and unstable economies of work) and are the key mobile component of the assemblage (Drahokoupil and Piasna 2017). Finally, customers (who access restaurants’ offerings through the Deliveroo app) have both a customer demand role, and locational and temporal agency as the generators of demand for food delivery.

### Platform urbanism in the Global South

Much of the focus of research on platform urbanism is likely to be targeted at the key role of technology corporates and financial firms. While this is useful, it is likely to replicate much existing critique of the smart city’s corporate characteristics (Hollands 2015; Luque-Ayala and Marvin 2015; Cardullo and Kitchin 2018) if the focus is simply on critiquing corporate interventions into platform urbanism *because* they are corporate. It is also likely to replicate the critique of privileging cities in the Global North (Luque-Ayala and Marvin 2015; McFarlane and Söderström 2017). A fruitful way forward would be to suggest that studies and critiques of platform urbanism shall focus on cities in the Global South where social, economic and geographical conditions are contrastingly different. Platform urbanism exists as much in cities and informal settlements in the Global South as it does in Manhattan or Singapore. In the case of mobilities platforms, for example, while Uber is based on cars (including self-driving vehicles) in the Global North, ride-hailing platforms in the Global South exhibit locally-engaged characteristics. Indonesian ride-hailing service Go-Jek, or Bangladeshi equivalent Pathao (পাঠাও), for example, focus (exclusively or in part) on motorbike-based ride sharing. This responds to material conditions whereby urban congestion means that motorcycle transport is attractive for urban mobility. At the same time, other platforms, such as fintech platforms, are key to the functioning of urban and national economies in the Global South. Fintech platforms are central to the penetration of mobile banking in African countries, for example: global consulting firm McKinsey estimates that there are 100 million African banking customers who access banking through smartphones, and that their transactions are worth US$2.1bn annually (Chironga et al 2017), while the significance of mobile banking and fintech platforms for a range of financial services from banking, to remittances, to loans, has been widely documented (Bettman and Harris 2014; Mohapatra and Ratha 2011). It is key for future research agendas on platform urbanism to also consider the implications of platforms for cities in the Global South. More specifically, the rootedness of platform urbanism in specific urban contexts leads to the questions of how and in what ways is platform urbanism affecting, or even reshaping, urbanism in the Global South.

### Platforms and emergent pandemic events

With platforms’ promise of near-real-time data-gathering, analytics, and response, it is often mobilized as a governance strategy to respond to crisis and uncertain events with large scale implications. It is therefore crucial to engage with the development of platform urbanism as a result of crisis and uncertain events affecting individual or multiple cities with mass effects. The Covid-19 pandemic is a case in point. It has spread globally during the time this paper was being written, and has occasioned the development of multiple platform-based responses. These responses have had, and promise to have, important implications. While a full consideration of the ramification of the pandemic and its effects on digital platforms and their urban applications falls outside the scope of this paper, the following provides some brief comments for how understanding platform urbanism may contribute to an understanding of unfolding pandemic events that are closely linked to algorithmic governance and digital intermediation.

The first emergent issue we wish to highlight is the link between the pandemic, urban governance, and citizenship. Multiple citizenship regimes have been explored in relation to the smart city (Joss et al. 2017): the Covid-19 pandemic has added to this by giving rise to what has been termed ‘pandemic citizenship’ (Calzada 2020): a form of citizenship characterised by shared fear, uncertainty, and risk. Secondly, while debates around smart urbanism focused in part on the deepening of digital inequalities that may result from urban life becoming ‘smarter’, the pandemic has highlighted how platformisation may become ever more prevalent, interlinked with multiple aspects of social life, and therefore open to mechanisms of state control and even coercion, and to the exercise of what has been called ‘datapower’ (Söderstrom and Mermet, 2020). There are questions to be raised, for example, about the increasing difficulty of ‘opting out’ of platform urbanism and the broader platform economy (Spangler 2020), as pandemic digital governance includes considering measures such as ‘voluntary’ vaccine passports that will unlock access to transport (especially air travel and international destinations) and which are recognised across multiple jurisdictions and borders. There have also been calls to use platform technologies in the context of pandemic urban governance, as a way of monitoring, recording and reporting compliance with pandemic measures (Basmi et al. 2021). These trends raise issues around: a) who is potentially excluded in platform-based pandemic governance, such as those who cannot or elect not to take vaccines, those who live in less wealthy countries where platform technologies are not a feasible current option, or citizens with less agency in specific contexts, such as internal migrants; and b) the bodily experience of platform-enabled pandemic urbanism (Bissell 2020), including the quotidian negotiations between platform urbanism’s actor networks and the citizen-as-consumer conjoined with the citizen-as-biopolitical-subject.

Finally, it is key to note that the platformisation of data and responses to the pandemic have also highlighted the gaps in the assumed ubiquity and coherence of platform urbanism. Urban platforms are most often viewed as a *cam*era *obscura* whose workings are complex, high-tech, and often protected by corporate and/or state *fiat*. At the same time, recent research has encouraged a view of platform urbanism as less than opaque (Fields et al. 2020). This dovetails with the recognition that platform-based responses to the pandemic have encountered numerous problems, obstacles and setbacks, not least in the most technologically advanced cities (Lai et al. 2020). Meanwhile, there has been a more grassroots and activist engagement with platforms during the pandemic, from the emergence of multiple local and wider-scale citizens’ networks, to support groups and activist interventions focused on the most vulnerable (Sòderstrom 2020). All these developments suggest that it is important to examine the unevenness, inequalities, struggles and conflicts embedded in urban platforms, through which alternative forms of platform urbanism may emerge.

### Imagining the future city

Platforms also change the way in which the city is imagined from an experiential point of view. Smartphone-based platforms, for example, enable a view of the city as a series of consumption and service pathways, where data and urban features that are relevant to consumption, mobility and governance are made prominent, and other areas of urban experience are left obscure. This raises questions about the extent to which ‘platform accumulation’, based on the generation and capture of torrents of data, also effectively functions to render specific parts of the city visible, while marginalizing others from the geospatial imaginary. This facet has been explored through work using spectral analysis of visual digital traces in urban environments (Leszczynski 2018). Boy and Uitermark (2017), for example, highlight how certain streets and neighbourhoods are rendered visible over and above others through users’ engagement with platforms such as Instagram. In this sense, the visualities associated with platform urbanism can be seen, after Rose and Willis (2018), to both learn about, and anticipate, the urban future. Platform technologies, then, become the way through which a specific form of digital ocularcentrism is mediated and centred on the city. This points to the notion that ‘the concept of the urban imaginary might require some rethinking in relation to cities where so much of everyday life is now digitally mediated’ (Rose 2018: 108). At the same time, there is the need to consider, in future research, the contingent nature of digital visualities associated with the city, where contingency is understood as the way in which platformization produces unstable, highly personalized, and continuously changing representations based not only on an aggregate target audience, but on individual recipients and users of urban representations.

Meanwhile, as planetary platformization is ongoing in reconfiguring every aspect of urban life, we also need to ask whether there are alternatives to dominant modes of deploying and organizing platform urbanism, as well as ways in which existing platforms are being subverted in cities across the Global South and North. The platform urbanism we have discussed in this paper emerges from the development of platform capitalism, and is an evolution of the smart city. But are platforms always capitalist? Could non-capitalist platforms lead to a different mode of platform urbanism, and therefore influence the ways we imagine about our urban future? These are also imperative questions for scholars of platform urbanism to answer.

## Data Availability

All data referenced in this article is publicly available.

## Abbreviations

AI: Artificial Intelligence
AWS: Amazon Web Services
GPS: Global Positioning System
ICT: Information and Communications Technology
NGO: Non-Governmental Organisation
OS: Ordnance Survey

## Footnotes

1 These categories are: a) advertising platforms, such as Google, based on data extraction from users, with the data being analysed for the purpose of selling advertising ‘space’; b) cloud platforms, such as Amazon Web Services (AWS), which focus on renting cloud-based software and hardware to corporates; c) industrial platforms, such as those developed by GE, and aimed at optimizing manufacturing; d) product platforms, such as Spotify, which use established platforms to ‘transform a traditional good into a service and by collecting rent or subscription fees on them; and e) lean platforms, such as Airbnb, which focus on reducing asset ownership and associated costs (Srnicek 2017, 49). This typology, as Srnicek specifically noted, is not intended as a fixed matrix used to explain platform capitalism. For example, a corporation may operate across different platforms.

2 As Bratton (2015, 44) explains using the example of Amazon:‘[I]ts platform logic is based on the massive coordination of pricing, retail display, storage, and delivery of its flat commerce ontology of objects. If Google’s mission is to organize the world’s information, Amazon’s may be to organize the world’s commodities.’ Following on from this, while platform urbanism is focused on the city, platforms themselves draw on multiple geographical scales in their development, deployment, use and regulation.
